# Social Determinants of Smoking Among Young Adults in Jordanian Universities

**DOI:** 10.7759/cureus.94839

**Published:** 2025-10-18

**Authors:** Abdullah Mousa, Ruby Abu Nassar, Muath Mohammad Dabas, Leen Aburumman, Omar Muhammad Alkhatatbeh, Rouba S Orabi, Ahmad Difallah Al Darawsheh, Zaid Alkayed

**Affiliations:** 1 School of Medicine, The University of Jordan, Amman, JOR; 2 Department of Psychiatry, School of Medicine, The University of Jordan, Amman, JOR

**Keywords:** family influence, jordan, peer influence, public health campaigns, smoking behavior, university students

## Abstract

Background: Smoking remains a major public health challenge worldwide. This study explores a key aspect and a significant contributor to the smoking habits of young adults who are university students, which is the influence of family and friends. This study helps to organize more targeted health campaigns for smoking cessation and provides a more in-depth look at the psychology of smokers.

Methods: This study employs a cross-sectional design. The questionnaire encompassed four sections: demographics, smoking history, reasons for smoking and quitting, and social and family influence.

Results: Of all the participants, 24.8% were identified as current smokers. The results showed that the strongest predictor linked to smoking was having smoking friends, with an odds ratio of 5.05 (p<0.001), followed by the family's behavior toward smoking, with an odds ratio of 3.53 (p<0.001). Additionally, around 40.0% of smokers reported being dependent. Only 15.2% of smokers reported a frequent exposure to public health campaigns.

Conclusion: The study demonstrates essential factors that contribute to smoking behaviors; one of them is friends who are smokers. Additionally, the lack of public health campaigns is a concern. Therefore, public health interventions targeting smoking cessation are recommended. Further studies can target the effect of public health interventions on the attitudes of smokers.

## Introduction

Smoking is a significant cause of preventable death and a primary risk factor for cancer, heart disease, and lung disease [1]. Over one billion people smoke worldwide, with 80% of smokers coming from developing countries, according to the World Health Organization (WHO). According to some estimates, tobacco use is going to cause the deaths of almost eight million people annually by 2030 [2]. Jordan has one of the highest rates of tobacco use in the region and the world, particularly among adolescents and adults, with cigarettes and hookah being the most commonly used forms [1]. Smoking typically starts in adolescence, which may lead to health decline in later adulthood [3].

At the international level, measures aiming at reducing the use of tobacco products are primarily geared towards adolescents and youths. There is a gap in knowledge concerning the context-specific determinants of smoking behavior, particularly amongst the youth population. Most adult smokers started smoking as teenagers or young adolescents, which is why it is essential to ascertain what factors contribute to youth smoking behavior, and how mitigation measures can be put in place to curb such behaviors [4]. In addition to the family environment and communication techniques, parental smoking practices have a significant impact on university students' smoking behavior. They can affect whether they decide to start or stop smoking.

According to studies, teenagers who have parents who are addicted to nicotine are more likely to engage in heavier smoking habits, and the risk rises with increased exposure time [5]. In addition to family influence, peers play an important role in smoking initiation during adolescence. Most adolescents report that they were with friends when they smoked their first cigarette [6].

In Jordan, social norms further influence the relationship between family, peers, and smoking behaviors. Understanding how these cultural factors interact with family and peer dynamics is essential for developing effective prevention and intervention programs tailored to the specific needs of Jordanian university students.

This cross-sectional study primarily aims to explore how family and peers influence smoking behavior (initiation and continuation) among university and college students in Jordan. Our secondary aim is to explore how this influence changes students’ knowledge about smoking risks and participation in smoking cessation programs. This will help shape public health campaigns and smoking cessation strategies.

Prior studies from Jordan and other middle-income countries have shown that both family and peer dynamics are critical determinants of smoking initiation and continuation [7]. However, very few studies have examined social determinants, such as family or peer supportiveness, or exposure to anti-tobacco campaigns [8,9].

This study provides evidence from a large, representative sample of 612 university students in Jordan, examining how family behavior and peer influence interact with demographic factors to shape smoking behaviors. Our findings show that having friends who smoke increases the chances of smoking fivefold, while family or peer attitudes supportive of smoking triple the likelihood of smoking. Moreover, the study highlights the low exposure of anti-smoking campaigns, showing a gap in public health interventions. These results contribute new knowledge to the literature by demonstrating how family and peer relationships strongly predict smoking among young adults in a lower-middle-income country context, such as Jordan. This has implications for encouraging youth- and culturally focused tobacco control strategies worldwide.

## Materials and methods

This cross-sectional study examines the impact of family and peers on smoking behaviors among university students in Jordan. Participants in the study include public and private Universities, as well as college students in Jordan, ranging from first-year students to those pursuing master's and doctoral degrees. A total of 612 students completed the questionnaire. Inclusion criteria were students aged 18-30 years who are currently enrolled in Jordanian universities. Exclusion criteria were incomplete questionnaires and participants who declined to give consent.

Participants were recruited through an open survey link distributed via official student groups and social media platforms (e.g., Facebook, Instagram, WhatsApp). The invitation message explained the study's purpose, outlined the eligibility criteria, assured confidentiality, and required participants to provide their consent. Students accessed the survey through a secure link and completed the questionnaire online. Furthermore, we aimed for a minimum sample size of 600 participants, calculated based on a 95% confidence level, a 5% margin of error, and an estimated smoking prevalence of 50% among university students to ensure sufficient power.

The questionnaire had four sections, which included demographics such as age, gender, city, university/college, year of study, and field of study.

To create a comprehensive measure of smoking intensity, the data collected from cigarettes, hookah, and vape use were standardized into cigarette equivalents based on existing research. Hookah usage was converted using the equivalence of 60 minutes of hookah smoking to 100-200 cigarettes [10], while vape use was transformed using the equivalence of 200 puffs (14.4-32.8 mg) to 13-30 cigarettes [11]. The total intensity variable was calculated as the sum of cigarette consumption, transformed hookah usage, and transformed vape puffs.

Smoking dependency was assessed using a composite index developed for this study. It was based on three binary indicators: feeling angry or stressed if unable to smoke for a long time, reporting inability to perform well on exams without smoking beforehand, and smoking immediately upon waking. Each item was coded as 0 (absent) or 1 (present), and their sum produced scores ranging from 0 to 3, corresponding to not dependent (0), mildly dependent (1), moderately dependent (2), and highly dependent (3).

Adherence to smoking restrictions was measured using three binary items reflecting compliance in different contexts: avoiding smoking in prohibited areas both inside and outside Jordan, avoiding smoking in banned areas inside Jordan only, and avoiding smoking in prohibited areas outside Jordan only. Scores ranged from 0 (non-adherent) to 3 (fully adherent), with intermediate values representing partial or mostly adherent behavior.

For data analysis, Python version 3.13 was utilized for data handling, cleaning, visualization, descriptive statistics, assumption checking, and model evaluation. Chi-square tests for associations between categorical variables and logistic regression analyses were performed using IBM SPSS Statistics for Windows, Version 27 (Released 2020; IBM Corp., Armonk, New York, United States). A significance level of 0.05 was set for all statistical tests.

In this study, ordinal variables were treated as continuous predictors in the logistic regression model to address multicollinearity and wide confidence intervals observed when using dummy encoding. Efforts to merge categories further worsened model fit and predictive accuracy. Box plots showed a monotonic increase in median probabilities across levels, and a linear trend in mean log-odds supported proportionality. Treating these variables as continuous improves model performance by reducing -2 Log-Likelihood, increasing accuracy, and narrowing confidence intervals, which aligns with the ordinal nature of the data and enhances interpretability.

The Institutional Review Board (IRB) of Jordan University Hospital (JUH) approved the study (Approval ID: 1472/2025/67). The study adhered to the tenets of Helsinki, ensuring adherence to ethical standards in design and ongoing monitoring. A brief description of the study’s aim was provided at the beginning of the questionnaire, and informed consent was obtained from all participants. Confidentiality was maintained throughout the study. Patients and the public were not involved in the design, conduct, reporting, or dissemination plans of this research.

## Results

Demographic characteristics and associations with smoking status

The study included 612 university students in Jordan, mostly aged 20-22 years (63.6%, n = 389), followed by 23-25 years (18.1%, n = 111), 17-19 years (15.2%, n = 93), and over 25 years (3.1%, n = 19). Female students represented 73.5% (n=450) and male students 26.5% (n=162). Most were in medical fields (74.7%, n = 457), with smaller proportions in scientific fields (14.2%, n = 87), literary fields (4.6%, n = 28), and humanitarian fields (4.1%, n = 25). Undergraduates comprised 94.4% (n = 578), and postgraduates 5.6% (n = 34).

Overall, 71.4% (n=437) reported never smoking, 24.8% (n=152) were current smokers, and 3.8% (n=23) were former smokers. Smoking prevalence increased with academic level, χ²(6, N = 612) = 13.72, p = .033, from 19.7% among first-year students to 47.1% among postgraduates. Age was significantly associated with smoking, χ²(3, N = 612) = 21.92, p < .001, with the prevalence increasing from 21.5% in the 17-19 age group to 63.2% in those over 25. Gender differences were significant, χ²(1, N = 612) = 38.7, p < .001, with 47.5% of male students and 21.8% of female students reporting smoking. Smoking also varied by field of study, χ²(4, N = 612) = 12.18, p = .016, with the highest rate in literary fields (46.4%) and the lowest in medical fields (25.1%). Demographics are presented in Table 1.

**Table 1:** Sociodemographic Characteristics by Smoking Status The total number of participants was 612, with 175 classified as current or former smokers and 437 as never smokers.

Sample Characteristics	Current or Former Smoker		Never Smoker		Chi-Square Value	p
	n	%	n	%		
Gender					38.69	< .001
Male	77	44	85	19.5		
Female	98	56	352	80.5		
Age Group					21.92	< .001
17 - 19	20	11.4	73	16.7		
20 - 22	99	56.6	290	66.4		
23 - 25	44	25.1	67	15.3		
More than 25	12	6.9	7	1.6		
Field of Study					12.18	.016
Medical Fields	115	65.7	344	78.7		
Scientific Fields	33	18.9	54	12.4		
Humanitarian Fields	9	5.1	16	3.7		
Literary Fields	13	7.4	15	3.4		
Others	5	2.9	8	1.8		
Academic Year					13.72	.033
First Year	12	6.9	49	11.2		
Second Year	18	10.3	34	7.8		
Third Year	28	16	88	20.1		
Fourth Year	63	36	180	41.2		
Fifth Year	15	8.6	28	6.4		
Sixth Year	23	13.1	40	9.2		
Post-Graduate	16	9.1	18	4.1		

Smoking behaviors, dependence, and cessation patterns 

**Figure 1:**
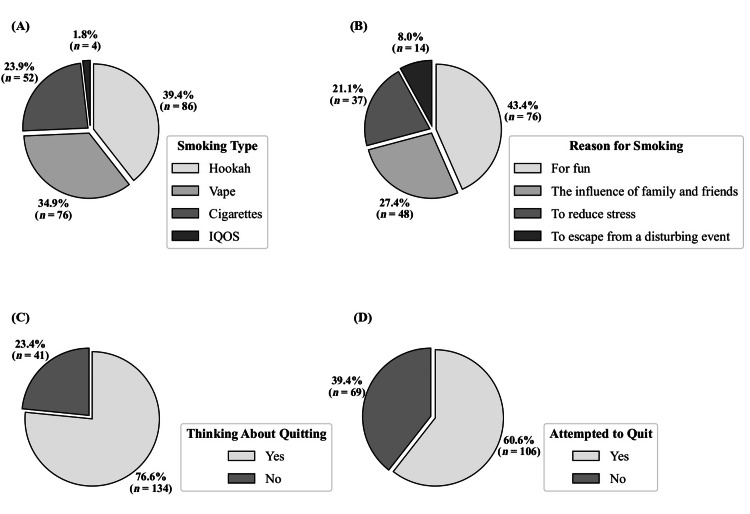
A Profile of Smoking Behaviors among Smokers: Types, Reasons, and Quitting Intentions (A) Smoking type. (B) Reasons for smoking. (C) Thinking about quitting. (D) Attempted to quit. For panel (A), percentages represent the proportion of responses for each smoking type, not the percentage of participants. Each participant could select multiple types of smoking.

**Figure 2:**
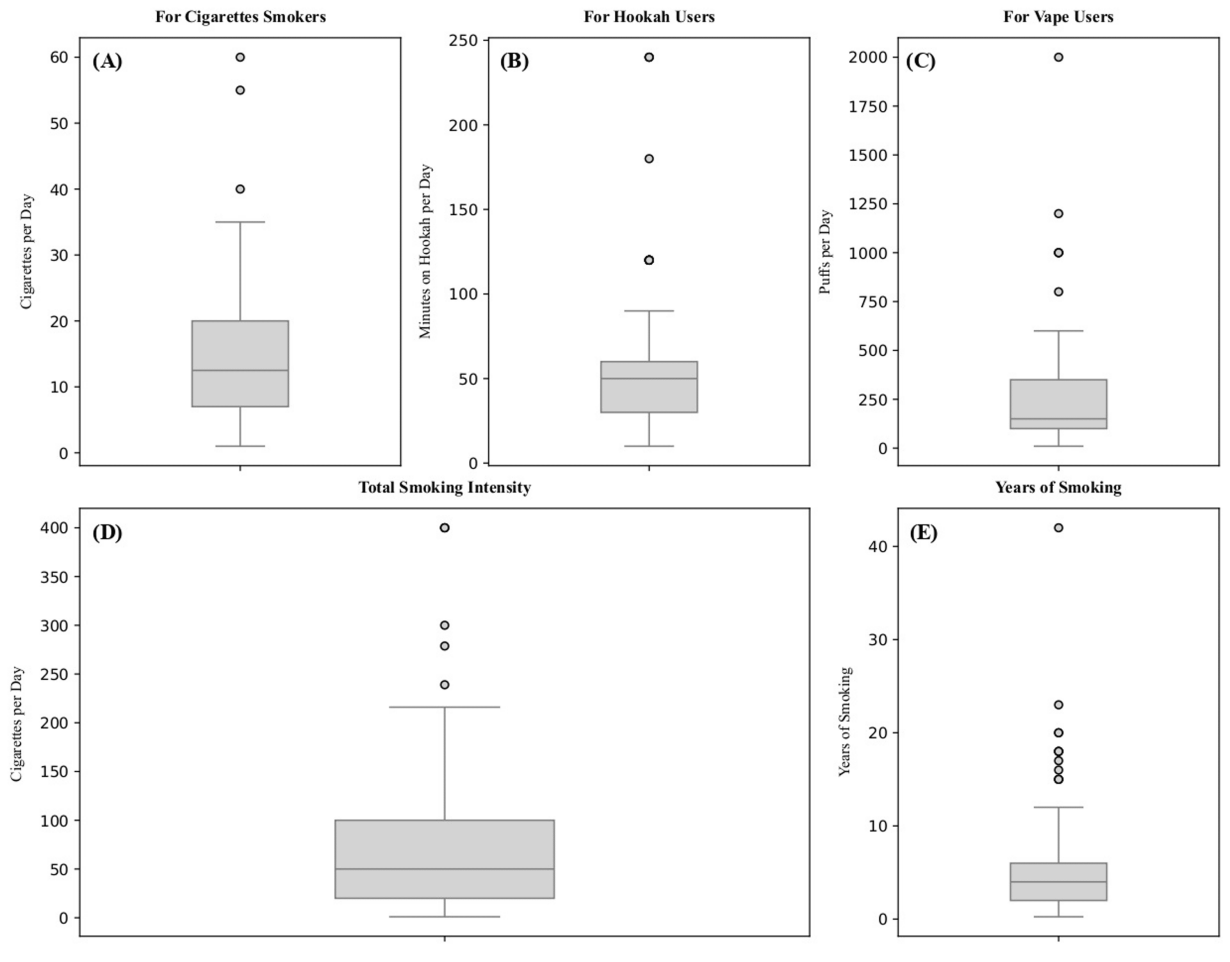
Daily Smoking Patterns: Cigarettes, Hookah, and Vape Use (A) Cigarettes smoked per day among cigarette smokers (n = 50). (B) Minutes of hookah per day among hookah users (n = 86). (C) Puffs per day among vape users (n = 76). (D) Total smoking intensity across all products. (E) Years of smoking.

Among smokers (n=175), hookah use was reported by 49.1% (n=86; Mdn duration=50 min/day, IQR=30-60), vaping by 43.4% (n=76; Mdn puffs/day=150, IQR=100-350), cigarettes by 28.6% (n=50; Mdn=12.5/day, IQR=6.75-20), and IQOS by 2.3% (n=4; Mdn=11/day, IQR=7-18). Poly-use resulted in cumulative percentages exceeding 100%. Median overall smoking duration was four years (IQR=2-6). Standardized intensity equaled a median of 50-cigarette equivalents/day (IQR=19.9-100) (Figures 1, 2).

Primary reasons for smoking included enjoyment (43.4%, n=76), family/peer influence (27.4%, n=48), stress relief (21.1%, n=37), and coping with distressing events (8.0%, n=14). Most smokers (76.6%, n = 134) expressed a desire to quit, and 60.6% (n = 106) had previously attempted to do so. Of these, 89.6% (n=95) tried independently, 7.5% (n=8) used nicotine replacement, and 2.8% (n=3) sought clinical support. Former smokers (n = 23) cited health concerns (30.4%, n = 7), family pressure (21.7%, n = 5), financial issues (21.7%, n = 5), religious/societal influence (21.7%, n = 5), and friends (4.3%, n = 1).

Dependence indicators included anger/stress when unable to smoke (30.9%, n=54), smoking immediately on waking (24.6%, n=43), and difficulty concentrating before exams without smoking (16.6%, n=29). Complaints about the smell were reported by 9.7% (n = 17) of the participants. Compliance with restrictions was mixed: 50.3% (n=88) avoided prohibited areas universally, 11.4% (n=20) complied only abroad, 10.3% (n=18) only within Jordan, and 40.6% (n=71) were non-adherent. Higher adherence was rare, with mostly adherent individuals (2.3%, n = 4) and fully adherent individuals (5.1%, n = 9). Dependency classification revealed 60.0% (n=105) as non-dependent, 18.9% (n=33) as mild, 10.3% (n=18) as moderate, and 10.9% (n=19) as high.

Public health campaigns, family, and social circles

**Figure 3:**
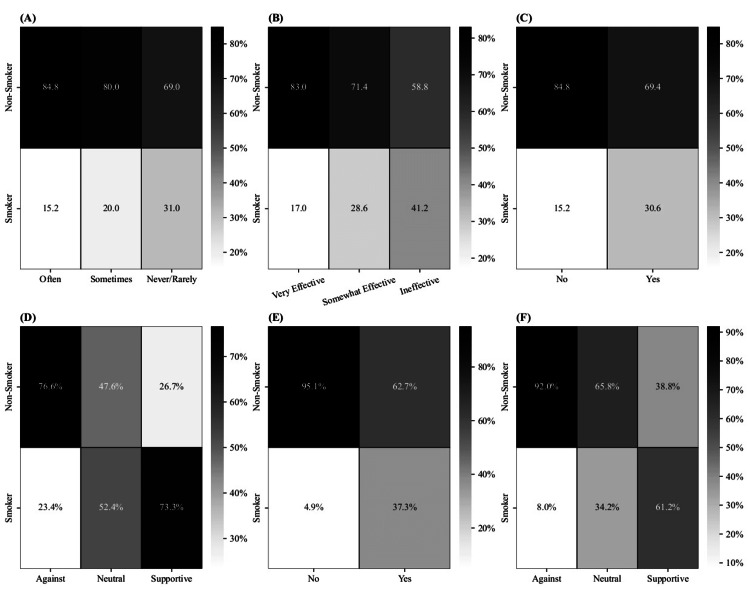
Proportions of Smoking Status across Various Social and Environmental Factors The figure presents six heatmaps illustrating the cross-tabulations between smoking status and six variables: (A) smoking status vs. frequency of exposure to public health campaigns about smoking, (B) smoking status vs. perceptions of the effectiveness of smoking cessation programs, (C) smoking status vs. family smoking status, (D) smoking status vs. family behavior toward smoking, (E) smoking status vs. friends' smoking status, and (F) smoking status vs. friends' behavior toward smoking. Each cell displays the percentage of participants within each smoking status group relative to the corresponding category of the variable being compared, with darker shades indicating higher percentages. The term "Smoker" includes both current smokers and former smokers.

Figure 3 summarizes the associations between smoking status and several social environmental factors. Most participants (80.7%, n=494) reported “never/rarely” being exposed to anti-smoking campaigns, 13.9% (n=85) “sometimes,” and 5.4% (n=33) “often.” Exposure was linked to smoking status, χ²(2, N = 612) = 7.36, p = .025, with the prevalence being highest in the “never/rarely” category (31.0%) and lowest in the “often” category (15.2%; Figure 3A). Regarding cessation program effectiveness, 66.8% (n=409) rated them “somewhat effective,” 17.3% (n=106) “very effective,” and 15.8% (n=97) “ineffective.” Smokers were more likely to perceive them as ineffective (41.2%) and less likely to perceive them as very effective (17.0%; χ²(2, N=612) = 14.6, p < .001; Figure 3B).

The majority (87.1%, n=533) reported relatives who smoke. Smoking prevalence was higher among those with relatives who smoked (30.6%) versus those without (15.2%; χ²(1, N=612)=7.98, p=.005; Figure 3C). Among these, smokers were commonly fathers (53.0%, n = 272), mothers (53.0%, n = 272), siblings (40.7%, n = 209), and second-degree relatives (66.3%, n = 340). Of 533 respondents, 51.6% received and accepted advice against smoking, 40.7% received none, and 7.7% dismissed advice. Prevalence varied significantly (χ²(2, N=533)=37.78, p<.001), lowest when no advice was given (16.6%) and highest when dismissed (53.7%). Family stance also mattered: 83.8% (n=512) opposed smoking, 13.7% (n=84) were neutral, and 2.5% (n=16) were supportive. Smoking prevalence was 73.3% in supportive families versus 23.4% in opposed (χ²(2, N=612)=44.78, p<.001; Figure 3D). Among smokers from opposed families (n=120), 66.7% faced refusal without punishment, and 33.3% were punished.

Most participants (73.2%, n=448) had smoking friends. Smoking prevalence was 37.3% with smoking friends versus 4.9% without (χ²(1, N=612)=61.72, p<.001; Figure 3E). Friends’ attitudes were neutral (44.4%, n=272), opposed (38.7%, n=237), or supportive (16.8%, n=103). Prevalence was highest among those with supportive friends (61.2%) and lowest among those with opposed friends (8.0%; χ²(2, N = 612) = 106.84, p < .001; Figure 3F). Peer pressure was minimal, with 83.0% (n = 508) reporting no peer pressure. Among non-smokers, 9.6% (n=42) credited peer influence for avoiding smoking. Peer pressure contributed to initiation in 32.9% of smokers (n = 50) and 39.1% of former smokers (n = 9), but quitting was attempted in only 13.0% (n = 3).

Predictors of smoking: logistic regression

Binary logistic regression (Table 2) showed excellent fit: Omnibus χ²(10)=229.68, p<.001; Hosmer-Lemeshow χ²(8)=12.75, p=.121; Nagelkerke R²=.45, explaining 45% of variance. Classification accuracy was 79.2%.

**Table 2:** Logistic Regression Analysis of Factors Associated with Smoking Status OR: Odds Ratio; C.I.: Confidence Interval. The ordinal independent variables included in the model are: frequency of exposure to public health campaigns (Exposure: 0 = Often, 1 = Sometimes, 2 = Rarely/Never), perceived effectiveness of smoking cessation programs (Effectiveness: 0 = Very Effective, 1 = Somewhat Effective, 2 = Ineffective), friends’ and family’s behavior toward smoking (0 = Against, 1 = Neutral, 2 = Supportive), and age group (0 = 17–19, 1 = 20–22, 2 = 23–25, 3 = More than 25). These variables were included as ordinal predictors and treated as continuous.

Variable	B	S.E.	Wald	p	OR	95% C.I.for OR	
						Lower	Upper
Exposure	0.64	0.24	7.11	0.008	1.90	1.18	3.03
Family History of Cancer (1 = Yes)	0.76	0.33	5.34	0.021	2.15	1.12	4.10
Effectiveness	0.65	0.21	9.84	0.002	1.92	1.28	2.88
Field of Study (Others vs. Medical)	0.47	0.25	3.54	0.060	1.61	0.98	2.63
Gender (Male vs. Female)	1.21	0.24	24.62	< .001	3.35	2.08	5.39
Friends' Behavior Toward Smoking	1.13	0.18	41.87	< .001	3.10	2.20	4.38
Family's Behavior Toward Smoking	1.26	0.24	27.52	< .001	3.53	2.21	5.67
Friend Smoking (1 = Yes)	1.62	0.43	14.22	< .001	5.05	2.18	11.73
Family Smoking (1 = Yes)	0.72	0.42	3.00	0.083	2.06	0.91	4.68
Age	0.30	0.16	3.51	0.061	1.35	0.99	1.85

The strongest predictor was having smoking friends (OR>5). Family and friend supportiveness each tripled the odds, and men were >3 times more likely to smoke than women. Low campaign exposure nearly doubled odds, as did perceiving cessation programs as less effective. Family history of cancer also nearly doubled the odds. Non-medical fields and older age showed marginal significance, while having a smoking family member was non-significant but showed a trend toward increased odds.

## Discussion

According to the WHO's latest report, Jordan is one of the only six countries worldwide where tobacco use is still rising. This aligns with the Ministry of Health (MOH) in Jordan, which showed concerning rates of tobacco smoking. Our study sample revealed a smoking percentage of 28.6%, which is lower than the previously estimated percentages of 36.3% and 35.0% [7,12,13].

Among male students, 47.5% were smokers, which is lower than the predicted values by the WHO (57.8%) [12], the Jordanian MOH (66.1%) [13], and another study (59.1%) [14]. This difference is likely due to our younger university sample compared to the peak prevalence in men aged 35-44. However, female students in our sample showed a significantly higher percentage of smokers compared to the WHO and Jordanian MOH estimates. In our study, 21.8% of female students were identified as smokers, whereas the WHO estimate [12] reported 13.4% of women as smokers, 17% in the Jordanian MOH [13], and 11.4% and 13% in other studies [7,15]. This is highly concerning since smoking prevalence among women remained almost constant throughout the years [16]. This is possibly caused by increased smoking acceptance among Arab conservative communities [17]. Additionally, promoting smoking as a sign of empowerment and self-care has contributed to this rise. Moreover, the changes in technology and lifestyle have increased women's exposure to different types of smoking and contributed to smoking initiation [18].

Smoking rates increased significantly throughout university years, which is consistent with previous national and international studies. This can be due to increased dependence among university students, who began smoking out of curiosity, and due to more prolonged exposure to older smokers, who influence the attitudes and behaviors of younger students [7,15,19-21].

Parallel to what was mentioned previously, smoking percentage was found to increase with age, which is suggested in previous national studies [7,14,15]. Studies from Portugal, Bosnia and Herzegovina, and the UAE also reported similar results [21-23].

Regarding the study field, students who major in literature have higher smoking rates than those who major in other fields, including medicine. Previous studies from Ethiopia, Portugal, Bosnia and Herzegovina, China, and Italy have similar findings [19-22,24,25].

Regarding smoking patterns, the median total intensity of smoking is 50 cigarettes per day. This number is alarming as it shows a rise in smoking intensity compared with previous national and international literature [7,21,25,26]. However, fortunately, most smokers were classified as non-dependent, unlike previous studies that revealed higher levels of dependence [7,27,28].

The most common reported reason for smoking was enjoyment. Other remarkable factors include family or peer influence, stress relief, and coping with distressing events. These findings are consistent with previous studies conducted in Ethiopia, Italy, and elsewhere [7,9,14,19,25,29].

The majority of smokers in our study population desired to quit smoking, but only 60.8% had tried to quit. These findings are consistent with previous studies conducted in Jordan and international studies [7,19,25,27,29].

The causes for quitting smoking varied, with health-related concerns being the most common motivation. Other causes included family pressures, financial constraints, religious or societal influences, and the impact of friends. Other studies have revealed similar findings [7,14,29]. Health-related concerns were the main reason for quitting smoking in Bosnia and Herzegovina and Italy [22,25].

Interestingly, most smokers showed partial adherence to smoking restrictions, including avoiding smoking in prohibited areas. Previous studies have yielded similar findings, where participants adhered to smoking restrictions and believed that further regulations should be implemented. Additionally, an Italian study showed that smokers found smoking restrictions helpful in protecting others' health. Furthermore, a study carried out in Italy reported that implementing smoking-free policies reduces smoking among this age group [25].

Public health campaigns play an important role in combating smoking. Although a previous national study showed that anti-smoking advertisements and education programs were clear and visible [28], a significant percentage of smokers reported “never/rarely” being exposed to campaigns. However, smokers were more likely to perceive these programs as “ineffective", indicating that those smokers are indeed exposed to these campaigns. Still, the continuous exposure rendered them insensitive to these campaigns. Moreover, a study in Portugal showed that people who quit smoking have higher knowledge of tobacco use compared to smokers and non-smokers, which further stresses the importance of these campaigns in spreading eye-catching and up-to-date advertisements [21].

Data analysis revealed interesting points regarding the family effect on smoking. Firstly, smoking was significantly associated with family smoking history, where individuals with smoking relatives are more likely to be smokers. Previous research studies indicate similar findings [7,9] and attributes these findings to secondhand smoke exposure and its effect on the onset of their smoking habits [15]. Secondly, smoking is most prevalent among those who dismissed the advice from their family members who smoked, believing that they lacked the credibility to advise them. Finally, family support of smoking is an essential factor. Smokers were most prevalent among participants with families supportive of smoking. A previous study explained this by the fact that these families may have encouraged and facilitated smoking [15]. A study in Portugal mentioned that smoking rates are higher in university students because parental control was lost when these students left their homes for education [21].

Additionally, friendships play a significant role in smoking behaviors, as smokers are more likely to be friends with smokers. Previous studies revealed that most smokers smoked for the first time with their friends [30]. In addition to that, smoking was more prevalent among participants whose friends supported smoking. Previous national research reported that most smokers were encouraged by their friends to start smoking [7,29]. A Portuguese study mentioned secondhand smoke as another possible cause [21].

However, the peer pressure effect varied among participants. Non-smokers described peer influence as being protective. Ex-smokers and current smokers attributed smoking initiation to it, similar to what previous national [9] and international [19,25] studies have found.

Multiple factors were identified as being associated with smoking behavior. The strongest factor was having friends who smoked, which increased the odds of tobacco use fivefold.

A threefold increase in smoking odds was associated with supportiveness of family, supportiveness of friends, and gender, where men are more likely to be smokers.

A twofold increase in smoking odds was identified with lower exposure to public health campaigns, lower perceived effectiveness of smoking cessation programs, and having a family history of cancer. Age and field of study showed marginal significance, and having a smoking family member was identified as a non-statistically significant but a potential factor.

These results are comparable to those indicated in previous national studies [7,12,14,15]. Studies in Ethiopia, Sudan, Bosnia and Herzegovina, and Finland also revealed similar factors [19,21,23,26]. They also showed other factors such as secondhand smoking, physical activity, and socio-economic status, which emphasize the importance of a multidisciplinary approach in fighting smoking.

These findings can inform future efforts aimed at reducing smoking. Policymakers must pay urgent attention to women and first-year students, as smoking is considered the first step of illicit drug use. Additionally, they must design well-tailored smoking control programs to fit young adults. Moreover, they should also provide other means of entertainment for the youth.

Our study is one of the few studies that comprehensively examines the dual influence of both peers and family on smoking behavior among Jordanian university and college students. Secondly, the large sample size of 612 participants supports the generalizability of the findings within this demographic. What adds more strength to the study is the use of statistical methods to identify significant predictors of smoking behavior.

Despite its strengths, the study has some limitations. The use of self-reported data increases the possibility of response bias, in which participants could misreport their smoking behaviors according to cultural norms. In addition, the cross-sectional design weakens the establishment of causality. In other words, while associations between variables were positive, the direction cannot be determined. Third, the predominance of medical students in the sample and the gender imbalance (more female students than male students) limit the generalizability of the findings to the broader Jordanian student population. Fourth, although we standardized hookah and vape use into cigarette equivalents, these conversions may not precisely capture actual smoking intensity, which could affect the accuracy of dependency assessments. Fifth, the reliance on voluntary online responses may introduce selection bias, as students with stronger opinions or personal experiences with smoking might have been more inclined to participate. Finally, several unmeasured factors, such as socioeconomic status, parental education, and mental health conditions, were not assessed but could also influence smoking behaviors.

Furthermore, smoking dependency and adherence to restrictions were assessed using self-developed composite indices rather than validated standardized tools. These measures were intended for descriptive purposes only, and their validity and reliability were not formally evaluated. Findings should therefore be interpreted with caution, and future research should employ or validate established scales to ensure their validity and reliability.

Despite these limitations, the study provides valuable insights into the role of family and peers in shaping smoking behaviors among Jordanian university and college students, offering a strong evidence base for designing culturally tailored public health interventions.

Viewing these points pulls many suggestions. Future studies should employ longitudinal designs to elucidate the causal relationships between family and peer influences and smoking behavior. Additionally, they should expand the sample to include more students from a broader range of institutions, which would help improve the generalizability of the findings. Qualitative methods should be employed to gain a deeper understanding of the factors influencing smoking behaviors. Moreover, future research must evaluate the effectiveness of smoking prevention and cessation programs that cater to the cultural and social needs of Jordanian society.

## Conclusions

This study shows that there are gaps in public health efforts targeting smoking cessation, with 80.7% of all participants reporting “never” or “rarely” being exposed to smoking cessation campaigns, and 41.2% of smokers view the campaigns as ineffective. Moreover, the study findings showed that having smoking friends plays a key role in predicting smoking behaviors, with 37.2% of smokers having smoking friends. In addition, family attitudes, which also significantly influenced smoking behaviors in predicting smoking behaviors, strengthening the effectiveness of the families and friends’ roles in smoking cessation, will have a good impact.

To enhance smoking cessation efforts, more work should focus on increasing the number and quality of public health campaigns. Universities should implement culturally tailored, youth-oriented anti-smoking campaigns directly on campuses, alongside strict enforcement of smoke-free policies. Peer-led initiatives can be effective, as students are more likely to respond to their colleagues. Families should also be engaged in awareness programs to reinforce anti-smoking attitudes at home. More studies should be conducted targeting the effects of public health interventions on the attitudes of smokers.

## Appendices

Questionnaire

https://drive.google.com/file/d/1HJA0yZyKf0ch3Uz1VER19TeG_ZnLVVfQ/view?usp=drivesdk
